# The Bacterium *Akkermansia muciniphila*: A Sentinel for Gut Permeability and Its Relevance to HIV-Related Inflammation

**DOI:** 10.3389/fimmu.2020.00645

**Published:** 2020-04-09

**Authors:** Jing Ouyang, John Lin, Stéphane Isnard, Brandon Fombuena, Xiaorong Peng, André Marette, Bertrand Routy, Meriem Messaoudene, Yaokai Chen, Jean-Pierre Routy

**Affiliations:** ^1^Infectious Diseases and Immunity in Global Health Program, Research Institute, McGill University Health Centre, Montréal, QC, Canada; ^2^Chronic Viral Illness Service, McGill University Health Centre, Montréal, QC, Canada; ^3^Chongqing Public Health Medical Center, Chongqing, China; ^4^Department of Microbiology and Immunology, McGill University, Montréal, QC, Canada; ^5^State Key Laboratory for Diagnosis and Treatment of Infectious Diseases, National Clinical Research Center for Infectious Diseases, Collaborative Innovation Center for Diagnosis and Treatment of Infectious Diseases, The First Affiliated Hospital, College of Medicine, Zhejiang University, Hangzhou, China; ^6^Department of Medicine, Faculty of Medicine, Cardiology Axis of the Québec Heart and Lung Institute, Laval University, Laval, QC, Canada; ^7^Institute of Nutrition and Functional Foods, Laval University, Laval, QC, Canada; ^8^Centre de Recherche du Centre Hospitalier de l’Université de Montréal (CRCHUM), Montréal, QC, Canada; ^9^Hematology-Oncology Division, Department of Medicine, Centre Hospitalier de l’Université de Montréal (CHUM), Montréal, QC, Canada; ^10^Division of Hematology, McGill University Health Centre, Montréal, QC, Canada

**Keywords:** *Akkermansia muciniphila*, epithelial gut damage, inflammation, microbial translocation, HIV

## Abstract

Gut dysbiosis, namely dysregulation of the intestinal microbiota, and increased gut permeability lead to enhanced inflammation and are commonly seen in chronic conditions such as obesity and aging. In people living with HIV (PLWH), several lines of evidence suggest that a depletion of gut CD4 T-cells is associated with gut dysbiosis, microbial translocation and systemic inflammation. Antiretroviral therapy (ART) rapidly controls viral replication, which leads to CD4 T-cell recovery and control of the disease. However, gut dysbiosis, epithelial damage and microbial translocation persist despite ART, increasing risk of developing inflammatory non-AIDS comorbidities such as cardiovascular disease, diabetes mellitus, liver steatosis and cancer. In addition to ART, an emerging research priority is to discover strategies to improve the gut microbial composition and intestinal barrier function. Probiotic interventions have been extensively used with controversial benefits in humans. Encouragingly, within the last decade, the intestinal symbiotic bacterium *Akkermansia muciniphila* has emerged as the “sentinel of the gut.” A lower abundance of *A. muciniphila* has been shown in diabetic and obese people as well as in PLWH. Interventions with high levels of polyphenols such as tea or diets rich in fruit, the antibiotic vancomycin and the antidiabetic drug metformin have been shown to increase *A. muciniphila* abundance, contributing to improved metabolic function in diabetic and obese individuals. We hypothesize that gut microbiota rich in *A. muciniphila* can reduce microbial translocation and inflammation, preventing occurrences of non-AIDS comorbidities in PLWH. To this aim, we will discuss the protective effect of *A. muciniphila* and its potential applications, paving the way toward novel therapeutic strategies to improve gut health in PLWH.

## Introduction

Gut microbiota is composed of a community of microorganisms gathered in the gastrointestinal (GI) tract. The number of micro-organisms is 1–10 times greater in the GI tract than the number of host cells in humans. Additionally, the number of microbial genes is 100 times greater than that of the human genome (1). In normal, healthy conditions, a state of eubiosis is attained when the composition of the gut microbiota is balanced. The gut microbiota is emerging as a prominent player in maintaining health through several metabolic and immune pathways. Dysregulation of gut microbiota composition, also known as dysbiosis, can be associated with gut barrier dysfunction and intestinal homeostasis disruption through translocation of microbial products and proinflammatory factors (2). Increasing evidence has put a spotlight on the contribution of gut dysbiosis and its related inflammation in obesity, diabetes mellitus (DM), cancer, aging and more recently, human immunodeficiency virus (HIV) infection (3–7).

In people living with HIV (PLWH), intestinal CD4 T-cells are a preferential target of the virus due to their high expression of CCR5, a chemokine co-receptor allowing for the entry of HIV, leading to their massive depletion during early infection (8, 9). This disruption in gut homeostasis results in dysbiosis, microbial translocation and systemic inflammation (10, 11). Antiretroviral therapy (ART) has transformed the lives of PLWH by rapidly controlling viral replication and allowing CD4 recovery, reducing morbidity and mortality. However, despite controlling viral load and CD4 T-cell count, long-term ART reduces but does not normalize gut dysbiosis, microbial translocation, immune activation and inflammation (12–14). In addition to HIV itself, coinfection with cytomegalovirus or viral hepatitis, leaky gut and microbial translocation also lead to inflammation which has been associated with the risk of non-AIDS comorbidities (13, 15–18). The direct influence of dysbiosis, microbiota by-products, epithelial barrier and local immune response will need further studies to define their distinctive role on systemic inflammation and subsequent development of non-AIDS comorbidities. Cardiovascular disease, DM, liver steatosis, neurocognitive disorders and cancer represent the most frequent manifestations of non-AIDS comorbidities, which represent a new frontier in the management of PLWH in today’s medical practice (19–21). Thus, in addition to ART, strategies to improve the gut microbial composition and intestinal barrier function are emerging as a research priority.

Converging evidence has recently demonstrated the key role of commensal bacteria harbored in the GI tract. Interestingly, the bacterium *Akkermansia muciniphila* has been described as a protective ally against the development of metabolic diseases and colitis (22). *A. muciniphila* of the phylum Verrucomicrobia, was first isolated and characterized in 2004. This Gram-negative, anaerobic, non-motile, non-spore-forming bacterium has been considered to be a next-generation beneficial microbe (23). In humans, *A. muciniphila* colonizes the intestinal tract in infanthood and will reach 1–4% of the fecal microbiota by adulthood (24–26). Furthermore, studies have shown a link between low *A. muciniphila* abundance and increased occurrence of inflammatory metabolic diseases such as diabetes, obesity, ulcerative colitis (UC) and Crohn’s disease (CD), all of which are associated with epithelial gut damage and high permeability (27–35). On the other hand, supplementation with *A. muciniphila* can help protect from specific metabolic disorders, inflammatory diseases and increase response to cancer immunotherapy (4, 36–43). Moreover, increasing *A. muciniphila* abundance with the antidiabetic drug metformin or with high polyphenol interventions such as tea or diets rich in fruit further improves metabolic function in diabetic and obese individuals (42, 44–50). The causal or consequential role of *A. muciniphila* in protection from various diseases in humans remains under debate. Some evidence points toward this symbiotic intestinal bacterium as an emerging “gatekeeper of the gut”, associated with gut barrier integrity and the regulation of inflammation (22, 51, 52). Herein, we discuss recent advances in the understanding of the protective effects of *A. muciniphila* and its potential relevance in HIV infection.

## The Multifunctional Properties of *A. Muciniphila*

*Akkermansia muciniphila* encodes a particularly wide repertoire of mucin-degrading enzymes in its relatively small genome, uses mucin as its sole source of carbon and nitrogen, and its downstream glycan byproducts can cross-feed other gut bacteria (23, 53, 54). Based on its unique properties, the bacterium was named after the Dutch microbial ecologist Antoon DL Akkermans for his contributions to the field (55). Additionally, this bacterium exhibits multiple biological functions, including promoting gut barrier integrity, modulating immune response, inhibiting inflammation and cross-feeding, called syntrophy, with other microbiota species.

The gut barrier is organized as a multi-layered and complex system which allows nutrient absorption while preventing the translocation of microbes and their products. Disruption of the gut barrier leads to the transit of luminal contents into the bloodstream, activating the immune response and inducing inflammation (56). Mucus covers the outer intestinal epithelial cell layer and serves as physical protection from penetration of micro-organisms and harmful compounds (57). In addition to degrading mucins, *A. muciniphila* was also found to stimulate mucin production (42, 52). In animal models, *A. muciniphila* supplementation increased the thickness of the colonic mucus layer approximately 3-fold, significantly more than the thickness increased induced by the beneficial bacterium *Lactobacillus plantarum* (52). Furthermore, *in vitro*, *A. muciniphila* was found to improve enterocyte monolayer integrity by binding directly to the enterocytes (51). Ottman et al. also showed that the outer membrane protein Amuc_1100 of *A. muciniphila* improved epithelial cell monolayer integrity in an *in vitro* culture after 24 h (58).

There is evidence to show that *A. muciniphila* may regulate inflammation. Supplementation of this bacterium attenuated inflammation in an accelerated aging mouse model (52). Other studies have also shown the anti-inflammatory properties of *A. muciniphila* in different mouse models including germ-free, liver injury and obesity models (59–64). Huck et al. (62) reported that *A. muciniphila* could reduce inflammation induced by *Porphyromonas gingivalis* in lean or obese mice. Ansaldoi et al. (59) demonstrated that *A. muciniphila* plays a context-dependent role in the induction of gut-resident T-cells during homeostasis in mice. Sessa et al. reported in a cross-sectional study of perinatally HIV-infected children and adolescents that *A. muciniphila* abundance was associated with elevated IL-6 and soluble CD14 (65).

Additionally, it should be noted that there are also other microbes which are commonly found in the mucus layer aside from *A. muciniphila*. These microbes include bacteria such as *Faecalibacterium prausnitzii*, *Eubacterium rectale*, *Roseburia intestinalis*, and *Anaerostipes caccae* which produce the anti-inflammatory short-chain fatty acid (SCFA) butyrate (66–69). Butyrate-producing bacteria do not have the ability to degrade mucus, but use carbon and nitrogen degraded by mucin-degraded species such as *A. muciniphila* (53). Belzer et al. (66) reported that coculturing *A. muciniphila* with non-mucus-degrading butyrate-producing bacteria *F. prausnitzii, A. caccae*, and *Eubacterium hallii* resulted in syntrophic growth and production of butyrate. Thus, not only does *A. muciniphila* play an important role by itself in protecting the gut epithelium, but also supports anti-inflammatory intestinal microbiota.

Due to this, and considering its relatively high abundance at all stages of life, *A. muciniphila* is considered a promising beneficial microbe for some diseases, including metabolic disorders and cancers.

## Supplementation of *A. Muciniphila* in the Context of Metabolic Disorders and Cancers

As a strictly anaerobic bacterium, culture of *A. muciniphila* needs to be conducted under strict conditions. Advances in the culture and preparation of *A. muciniphila* have made it feasible for study as a beneficial microbe (36, 70). Supplements of this promising bacterium include live *A. muciniphila*, pasteurized (killed) *A. muciniphila* and *A. muciniphila*-derived extracellular vesicles (AmEVs) (4, 36, 38).

Obesity and metabolic disorders including DM are closely associated with low-grade inflammation and intestinal dysbiosis (71). Everard et al. reported that the abundance of *A. muciniphila* was 3,300-fold lower in obese mice than in their lean littermates. A 4-week oral gavage of live *A. muciniphila* in mice reversed high-fat diet-induced metabolic disorders, including fat-mass gain, metabolic endotoxemia, adipose tissue inflammation, and insulin resistance, and increased intestinal levels of endocannabinoids that controlled inflammation, increased gut mucus, and increased expression of gut antimicrobial peptides such as regenerating islet-derived 3-gamma (Reg3γ) for innate immunity (40). In addition, even when *A. muciniphila* is killed through pasteurization, supplementation demonstrated beneficial effects by protecting from ovariectomy-induced fat mass gain (72). In overweight insulin-resistant humans, a randomized, double-blind, placebo-controlled pilot study showed that daily oral supplementation of 10^10^ live or pasteurized *A. muciniphila* bacteria for 3 months was safe and well tolerated, and improved insulin sensitivity, reduced insulinemia, plasma total cholesterol, body weight, fat mass and hip circumference, without great changes in the overall gut microbiota composition (43). Furthermore, AmEV administration was reported to enhance tight junction function, reduce body weight gain and improve glucose tolerance in high-fat diet (HFD)-induced diabetic mice, suggesting that derivatives of the bacterium are sufficient to induce a protective response (38). These findings suggest the direct benefit of this bacterium on the gut barrier and the host metabolism.

Remarkably, the influence of the gut microbiota composition in modulating tumor responses to immunotherapy has also been reported in various cancers such as melanoma, lung and kidney cancer. This effect was observed in different geographic regions where microbiota might differ (North America, Europe, East Asia) (4, 73–75). Reconstitution of germ-free mice with fecal material from lung cancer immunotherapy responders led to increased T-cell responses, and greater efficacy of anti-PD-1 therapy (4). Oral supplementation with live *A. muciniphila* after fecal microbiota transplantation (FMT) with non-responder feces restored the efficacy of PD-1 blockade in murine models (4).

Although the long term effects of *A. muciniphila* supplementation are unknown with concerns over the translocation of probiotics (76), this bacterium may play a crucial role in increasing the efficacy of metabolic and cancer therapies and provide strong scientific rationale to launch microbiota-based clinical trials.

## Strategies to Increase the Abundance of *A. Muciniphila*

Supplementation of *A. muciniphila* may be difficult or costly, however, strategies to indirectly increase the abundance of *A. muciniphila* exist through dietary interventions, the antidiabetic drug metformin, selective antibiotics and FMT.

Dietary polyphenols are natural antioxidants, which may help protect obligate anaerobes by scavenging oxygen radicals. Gurley et al. reported that administration of green tea to mice, with comparable levels of polyphenols to those consumed by humans, resulted in significant modulation of gut microflora, with the greatest increases observed in *A. muciniphila* (47). Concord grape, cranberry and the Amazonian fruit Camu Camu have been reported to increase the abundance of *A. muciniphila* in the intestinal tract approximately 7-fold, 15-fold, and 5-fold, respectively, reduced inflammation and body weight gain, and increased gut barrier integrity in obese mouse models (44, 45, 49). Although a currently unpopular option, caloric restriction such as intermittent fasting has shown increases in *A. muciniphila* abundance (77). To scale up this approach, diet-mimetic medications are under intense scrutiny. Among the most commonly used in both animals and humans is the anti-diabetic drug metformin.

Metformin is the most commonly used drug to treat DM2 and recently has been shown to reduce inflammation, exert anti-aging effects and modify the gut microbiota composition (78). Although metformin acts primarily as a glucose mediator in the liver by inhibiting hepatic gluconeogenesis, accumulating evidence suggests that metformin also mediates changes in gut microbiota composition (79–81). Convergent reports showed that metformin significantly increased *A. muciniphila* abundance in animal models (42, 46, 82). The nitrogen-rich structure of metformin may also play a role in the nurturing of *A. muciniphila*, which requires nitrogen for proliferation and survival (80). Thus, the use of metformin is a strategy to enrich the abundance of *A. muciniphila* in the gut, among its other metabolic benefits as seen in DM2 (83).

*A. muciniphila* is resistant to vancomycin, metronidazole, and penicillin (84). Selective antibiotic treatment with vancomycin was shown to dramastically increase *A. muciniphila* abundance in young non-obese diabetic (NOD) mice, reducing their glucose levels and the diabetes incidence when compared with untreated NOD mice (85). In two patients from the intensive care unit of Marseille, France, broad spectrum antibiotics increased *A. muciniphila* abundance to more than 40% in stools, without inducing gastrointestinal disorders (84). Furthermore, Uribe-Herranz et al. reported that in pre-clinical models to study the immune-based off-target (abscopal) effect of radiotherapy, oral supplementation with vancomycin increased *A. muciniphila* which was associated with tumor growth inhibition in mouse models (86). Although further explorations are required in humans, vancomycin treatment appears safe and able to increase *A. muciniphila* abundance in the gut microbiota.

Fecal microbiota transplantation is also effective in restoring eubiosis in colitis and metabolic diseases. Zhang et al. showed that transplanting fecal bacteria from people with normal glucose tolerance into DM2 mice downregulated levels of fasting blood glucose, postprandial glucose, total cholesterol, triglyceride, and low-density lipoprotein-cholesterol and increased the abundance of *A. muciniphila* (87). Huang et al. reported that FMT improved gastrointestinal symptoms and alleviated depression and anxiety in irritable bowel syndrome (IBS) patients. Further, gut microbiota analyses revealed that *Methanobrevibacter* and *A. muciniphila* were the most abundant fecal microbiota a month after compared to before FMT (88).

These animal models and human epidemiological studies suggest methods to increase *A. muciniphila* abundance in humans, but efforts to scale up its abundance in PLWH, and in turn improving their gut health and various metabolic factors, are yet unexplored (89).

## Leaky Gut and Dysbiosis in PLWH

HIV infection is characterized by a rapid decline in CD4 T-cell count, early gut mucosal damage, and subsequent translocation of microbial products through the now more permeable epithelium (10, 90). Circulating levels of lipopolysaccharide (LPS) and (1→3)-β-D-Glucan (BDG) are two clinically significant markers that assess the level of bacterial and fungal translocation, respectively, of which high levels lead to metabolic endotoxemia (89). Our group and others have shown that LPS and BDG translocation are correlated with immune dysfunction in PLWH and increased risk of non-AIDS comorbidities (91–94). Moreover, we and others have evaluated circulating intestinal fatty acid binding protein (I-FABP) and regenerating islet-derived protein-3α (REG3α) as two gut damage markers in PLWH (14, 95). I-FABP, an intracellular protein constitutively expressed in enterocytes, is released upon cell death and subsequently detected in the blood (96, 97). REG3α is an antimicrobial peptide secreted by intestinal Paneth cells into the gut lumen and upon gut damage, translocates into the blood (14). We observed that these two gut damage markers were correlated with HIV disease progression, microbial translocation and immune activation in PLWH (14). These findings point to the leaky gut as a significant contributor to chronic inflammation and non-AIDS comorbidities in PLWH.

Recently, accumulating evidence has suggested that the gut microbiota is emerging as a prominent player in the regulation of host metabolism and chronic inflammation (98, 99). Bacterial communities residing in the intestine of HIV-infected individuals have been shown to differ from those of individuals not infected with HIV, independently of age, sex and sexual practice (6). Dysbiosis is associated with impaired intestinal barrier activity, impaired mucosal immunity function and worse clinical outcome in PLWH (6, 16, 100, 101). Moreover, *A. muciniphila* was significantly depleted in ART-naïve and ART-treated PLWH, compared to uninfected controls (101, 102). In one study, Mutlu et al. demonstrated that PLWH had significantly less *A. muciniphila* abundance regardless of ART, CD4 count or viral load, compared to healthy controls (102). Rocafort et al. confirmed and expanded these results by showing that *A. muciniphila* abundance was significantly higher in 49 recently infected PLWH and 55 healthy controls compared to 71 chronically infected untreated PLWH. Furthermore, in 27 chronically infected ART-treated PLWH, *A. muciniphila* abundance was similar to healthy controls (101). These findings suggest that chronic HIV infection leads to progressive depletion of *A. muciniphila* abundance, and following ART initiation, *A. muciniphila* abundance returns to levels similar to those of healthy controls. The causative role of *A. muciniphila* abundance in HIV infection with respect to gut integrity and inflammation needs to be further elucidated.

## Hypothesis: *A. Muciniphila* as a Sentinel for Gut Permeability in PLWH

**FIGURE 1:**
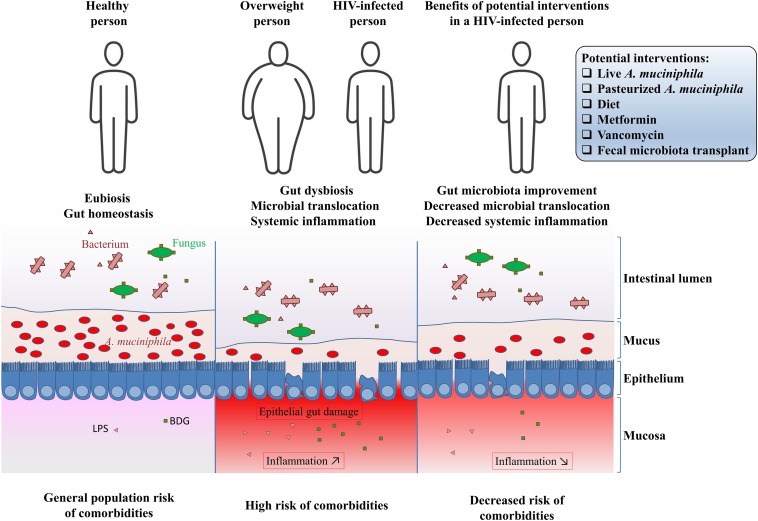
Potential interventions to increase *Akkermansia muciniphila* abundance in people living with HIV.

HIV infection, metabolic disorders and cancer share common features such as chronic inflammation and dysbiosis, which includes the decreased abundance of *A. muciniphila* in the gut microbiota (4, 40, 71, 101–104). Given this decreased abundance of *A. muciniphila* in PLWH, and considering the benefits of increasing *A. muciniphila* abundance in obesity, we hypothesize that *A. muciniphila* can act as a shield for gut permeability, preventing microbial translocation and reducing inflammation, with the aim toward decreasing risks of developing non-AIDS comorbidities in PLWH. Potential interventions that may increase *A. muciniphila* abundance in people living with HIV are shown in Figure 1.

Leaky gut has been considered one of the most important factors for microbial translocation and increased inflammation in PLWH (15). In three *in vitro* human cell line models, Caco-2, HT-29, and TIGK, *A. muciniphila* was reported to improve enterocyte monolayer integrity and increase the expression of cell–cell adhesion and tight junction molecules (51, 62). Furthermore, in an accelerated aging mouse model, the thickness of the colonic mucus layer increased approximately 3-fold after long-term *A. muciniphila* supplementation (52). Therefore, we propose that *A. muciniphila* might decrease inflammation by preserving gut barrier integrity and subsequently preventing microbial translocation in PLWH.

Furthermore, in PLWH, there is a lower abundance of butyrate-producing bacteria (105, 106). Butyrate plays an important role as an energy source for colonic epithelial cells and epithelial barrier integrity, T-cell activation, colonic regulatory T cell differentiation, gut and blood antigen presenting cell (APC) modulation (105–109). Lower abundance of butyrate-producing bacteria has been associated with poor clinical outcome in Crohn’s disease, ulcerative colitis and colon cancer (110, 111). Interestingly, *A. muciniphila* could promote butyrate-producing bacteria growth and butyrate production (66). We therefore suggest that *A. muciniphila*, by supporting butyrate-producing bacteria, may also decrease inflammation in PLWH through this method (106, 109).

Moreover, antimicrobial peptides in the gut play a prominent role as host defense effector molecules. Specifically, the C-type lectin REG3α secreted by human Paneth cells and its mouse ortholog REG3γ can bind peptidoglycan and serve as bactericidal agents against Gram-positive species (112). Live *A. muciniphila* supplementation showed an increased expression of the murine homolog REG3γ in an obese mouse model (40). Moreover, *A. muciniphila* was reported to induce immunoglobulin G1 (IgG1) antibodies, antigen-specific T-cell responses and intestinal adaptive immune responses (59). Therefore, *A. muciniphila* may improve intestinal homeostasis through the increased expression of REG3α in Paneth cells and inducing intestinal adaptive immune responses in PLWH.

## Conclusion

Epithelial gut damage, microbial translocation and inflammation are considered common determinant mediators of inflammatory non-AIDS comorbidities in PLWH. *A. muciniphila* has emerged as the “sentinel of the gut” and has been shown to promote gut barrier integrity, modulate immune response, inhibit inflammation and enrich butyrate-producing bacteria. Supplementation of *A. muciniphila* and other strategies promoting the abundance of *A. muciniphila* have been proven to be effective in some metabolic disorders and cancer. Recently, clinical trials involving metformin (113), prebiotics (CIHR/CTN NCT04058392) or FMT to increase *A. muciniphila* abundance have come into fruition, and we suggest that a gut microbiota enriched in *A. muciniphila* can reduce microbial translocation and inflammation, lowering the risk of developing non-AIDS comorbidities and improving quality-of-life in PLWH.

## Author Contributions

JO and JL wrote the first draft of the manuscript. SI, BF, XP, AM, BR, and MM provided critical revision of the manuscript. YC and J-PR conceived and designed the manuscript. All authors approved it for publication.

## Conflict of Interest

The authors declare that the research was conducted in the absence of any commercial or financial relationships that could be construed as a potential conflict of interest.
